# A comparison of taxon-like schizotypal clusters of non-clinical individuals on polygenic scores for schizophrenia and related phenotypes

**DOI:** 10.1192/j.eurpsy.2023.1884

**Published:** 2023-07-19

**Authors:** M. Alfimova, N. Kondratyev, V. Plakunova, T. Lezheiko, V. Golimbet

**Affiliations:** 1 Clinical Genetics Laboratory, Mental Health Research Center, Moscow, Russian Federation

## Abstract

**Introduction:**

Schizotypy is conceptualized to be on the continuum of the risk for psychosis. However, previous studies that used the dimensional approach to schizotypy failed to confirm the expected relations between schizotypal traits and polygenic risk scores (PRS) for schizophrenia. A taxonic approach is an alternative way of looking at schizotypy, but to the best of our knowledge it has not been used to explore the genetic architecture of this construct.

**Objectives:**

The present study aimed to fill this gap by comparing groups with different profiles of schizotypal traits on PRS for schizophrenia and related phenotypes.

**Methods:**

To find clusters with different combinations of schizotypal traits, we conducted data mining of an ethnically homogeneous cohort of 1377 healthy individuals completed the Schizotypal Personality Questionnaire. Four clusters emerged, termed low, positive, negative, and high mixed schizotypy. Approximately equal groups from each cluster were selected for genotyping. After quality control, PRS for schizophrenia, depression, neuroticism, and educational attainment were calculated for 320 individuals (mean age = 31.74, SD 12.25 years, 59% women) based on the summary statistics of the largest genome-wide association studies of the respective traits. The groups from different clusters were then compared on PRS.

**Results:**

The schizotypy groups were similar in age and sex composition but differed in educational attainment and neuroticism (as measured by the Eysenck Personality Inventory), with the high mixed schizotypes having the lowest education and highest neuroticism scores among the schizotypy groups. There were no statistically significant differences between the groups in any PRS. However, the high mixed schizotypy group showed the largest PRS for schizophrenia, neuroticism, and depression. At a nominally significant level, it differed from negative and positive schizotypes in schizophrenia PRS and from low schizotypy in neuroticism PRS. The positive and negative schizotypal groups had non-significantly lower schizophrenia PRS than low schizotypy subjects.

**Conclusions:**

Our study showed no reliable difference between groups with different schizotypal profiles in PRS of schizophrenia and related phenotypes. At the same time, a number of trends were observed suggesting that only the high mixed schizotypy might be viewed as a condition with an elevated genetic risk of schizophrenia. This is in line with Meehl’s quasi-dimensional model of schizotypy and warrants further investigation in larger samples. This work was supported by the Russian Foundation for Basic Research under Grant 20-013-00230.

**Disclosure of Interest:**

None Declared

